# Supplementary material for: Risk factors and high-risk subgroups of severe acute maternal morbidity in twin pregnancy: A population-based study
Source: PLoS One. 2020 Feb 28;15(2):e0229612. doi: 10.1371/journal.pone.0229612 (PMC7048407; doi:10.1371/journal.pone.0229612)
Supplement: S1 Table — (DOC) [file pone.0229612.s001.doc]

**S1 Table. EPIMOMS Multicriteria Standardized Definition of Severe Acute Maternal Morbidity, Developed Through a National Delphi Formal Expert Consensus Process**

| **ITEMS** | CRITERIA (AT LEAST ONE) |
| --- | --- |
| **Major obstetric bleeding** | . Volume of postpartum blood loss ≥ 1500 ml |
|  | . Blood transfusion ≥ 4 units RBC |
|  | . Uterine arteries embolization |
|  | . Vascular ligation, compressive uterine sutures |
|  | . Emergency peripartum hysterectomy |
| **Eclampsia** | Seizures in a woman diagnosed with preeclampsia, and if not, not attributable to another cause |
| **HELLP syndrome** | HELLP syndrome* only if associated with hepatic haematoma or rupture  *Haemolysis, Elevated Liver enzymes > 3 times the normal level and Low platelets < 50 000 |
| **Severe preeclampsia** | Preeclampsia** only if it induced a preterm delivery for a main maternal indication before 32 gestational weeks ** Defined as hypertension ≥ 140/90 and proteinuria ≥ 0.3 g/24 h |
| **Pulmonary embolism** | Clinical symptoms consistent with pulmonary embolism + confirmation with imaging + treatment (imaging: computed tomography or ventilation/perfusion scintigraphy or Doppler; treatment: heparin or thrombolysis or embolectomy) |
| **Placental abruption** | Placental abruption associated with a haematological dysfunction criterion |
| **Stroke** | Cerebral imaging showing cerebral infarction or haemorrhage, or venous thrombosis, or sub-arachnoid haemorrhage |
| **Cerebral transient ischaemic attack** | Neurological deficit with symptoms§ completely regressive in 24 hours and normal cerebral imaging, in absence of associated migraine confirmed by neurologist § Monocular blindness, aphasia, haemianopsia, motor and/or sensory uni- or bilateral disorders |
| **Severe psychiatric disorder** | . Severe acute psychiatric disorder or acute decompensation of chronic disease (psychosis, major depression, bipolar disorder) diagnosed by psychiatrist . Suicide attempt |
| **Cardiovascular dysfunction** | . Cardiac arrest . Acute pulmonary oedema with hypoxaemia < 60 mm Hg or SaO_2_ < 90% or treated with diuretics . Shock: Systolic blood pressure < 90 mm Hg during > 60 min or unresponsive to plasma expansion . Acute left ventricular dysfunction: left ventricular ejection fraction < 40% . Need for continuous IV vasopressor/inotrope drugs . Decompensation of a pre-existing cardiopathy with need for specialised management . Troponinaemia > 1 microg/l |
| **Respiratory dysfunction** | . Acute hypoxaemia < 60 mm Hg or SaO_2_ < 90% with spontaneous ventilation . Mechanical ventilation, or non-invasive ventilation in the absence of chronic disease, not related to anaesthesia |
| **Renal dysfunction** | . Acute renal failure with creatininemia > 135 µmol/l . Acute oliguria < 500 ml/24 h |
| **Neurological dysfunction** | Coma, regardless of stage and duration # # Stage 1 coma = impaired consciousness with obnubilation and reaction to painful stimuli only |
| **Hepatic dysfunction** | . Prothrombin time < 60%, in the absence of constitutional deficiency . Direct bilirubinaemia > 20 micromole/l |
| **Haematological dysfunction** | .Thrombocytopoenia < 50 000 /mm^3^ in the absence of chronic disorder . Acute anemia < 7 g/dl . Disseminated intravascular coagulation: platelets < 50 000/ mm^3^ or prothrombin time < 60% or fibrinogen < 2 g/l |
| **Emergency surgery apart from childbirth procedure** | . Secondary hysterectomy . Laparotomy for post-delivery complication apart from haematoma or parietal infection |
| **Admission to Intensive Care Unit** | |
| **Maternal death** |  |
