# Supplementary material for: Risk factors and high-risk subgroups of severe acute maternal morbidity in twin pregnancy: A population-based study
Source: PLoS One. 2020 Feb 28;15(2):e0229612. doi: 10.1371/journal.pone.0229612 (PMC7048407; doi:10.1371/journal.pone.0229612)
Supplement: S2 Table — * pregnancy: delivered at or after 22 weeks of gestation ** 95% confidence interval (CI) 5.6–6.6 *** nonexclusive categories. (DOC) [file pone.0229612.s002.doc]

**S2 Table. Incidence, timing, and underlying causal conditions of severe acute maternal morbidity in twin pregnancies in the JUMODA cohort**

|  | **Overall**  **n=8823**  **n** | **Rate of SAMM**  **per 100 twin pregnancies*** | **Proportion of SAMM cases**  **%** |
| --- | --- | --- | --- |
| **All SAMM** | 542 | 6.14** | 100.0 |
| **Underlying causal conditions of SAMM***** |  |  |  |
| Severe postpartum haemorrhage | 420 | 4.76 | 77.5 |
| Hypertensive complications | 54 | 0.61 | 10.0 |
| Abruptio placentae | 2 | 0.02 | 0.4 |
| Eclampsia | 16 | 0.18 | 3.0 |
| HELLP syndrome | 24 | 0.27 | 4.4 |
| Severe preeclampsia | 17 | 0.19 | 3.1 |
| Pulmonary embolism | 14 | 0.16 | 2.6 |
| Abruptio placentae without hypertension | 12 | 0.14 | 2.2 |
| Stroke or cerebral transient ischemic attack | 2 | 0.02 | 0.4 |
| Severe psychiatric disorders | 4 | 0.05 | 0.7 |
| Cardiovascular dysfunction | 10 | 0.11 | 1.8 |
| Respiratory dysfunction | 5 | 0.06 | 0.9 |
| Renal dysfunction | 28 | 0.32 | 5.2 |
| Hematological dysfunction | 87 | 0.99 | 16.1 |
| Neurological dysfunction | 0 | 0.00 | 0.0 |
| Emergency surgery | 42 | 0.48 | 7.7 |
| Death | 1 | 0.01 | 0.2 |
| Admission to an intensive care unit | 121 | 1.37 | 22.3 |

* pregnancy: delivered at or after 22 weeks of gestation

** 95% confidence interval (CI) 5.6-6.6

*** nonexclusive categories
