# Supplementary material for: Risk factors and high-risk subgroups of severe acute maternal morbidity in twin pregnancy: A population-based study
Source: PLoS One. 2020 Feb 28;15(2):e0229612. doi: 10.1371/journal.pone.0229612 (PMC7048407; doi:10.1371/journal.pone.0229612)
Supplement: S3 Table — (n = 8804 women). RR, relative risk; CI, confidence interval *Each relative risk is adjusted for all other variables in the table, multilevel multivariate Poisson regression model, with imputed data. (DOC) [file pone.0229612.s003.doc]

**S3A Table. Risk factors for severe acute maternal morbidity in twin pregnancies, sensitivity analysis after exclusion of women with severe acute maternal morbidity only due to hypertensive complication (19 cases), JUMODA cohort (n=8804 women)**

|  | |  | **At the beginning of pregnancy** | **At the end of pregnancy** |
| --- | --- | --- | --- | --- |
| **Potential risk factors** | | **Crude RR** | **Adjusted RR** | **Adjusted RR*** |
|  |  | **(95% CI)** | **(95% CI)** | **(95% CI)** |
| Maternal age (years) | |  |  |  |
|  | <30 | Reference | Reference | Reference |
|  | [30-35[ | 1.0 (0.9-1.3) | 1.0 (0.9-1.3) | 1.0 (0.9-1.2) |
|  | [35-40[ | 0.8 (0.6-1.0) | 0.8 (0.6-1.0) | 0.7 (0.6-1.0) |
|  | ≥40 | 1.7 (1.3-2.3) | 1.3 (0.9-1.9) | 1.2 (0.9-1.7) |
| Body mass index before pregnancy (Kg.m-2) | | |  |  |
|  | <18,5 | 1.0 (0.8-1.5) | 1.0 (0.8-1.5) | 1.1 (0.8-1.6) |
|  | [18.5-24.9] | Reference | Reference | Reference |
|  | [25-29.9] | 0.9 (0.7-1.1) | 0.9 (0.7-1.1) | 0.8 (0.7-1.1) |
|  | [30-34.9] | 0.8 (0.5-1.1) | 0.8 (0.5-1.1) | 0.7 (0.5-1.0) |
|  | ≥35 | 0.7 (0.4-1.2) | 0.8 (0.4-1.4) | 0.7 (0.4-1.2) |
| Country of birth | |  |  |  |
|  | Europe | Reference | Reference | Reference |
|  | North Africa | 1.2 (0.9-1.5) | 1.3 (1.0-1.7) | 1.3 (1.0-1.7) |
|  | Sub-Saharan Africa | 1.3 (0.9-1.8) | 1.6 (1.1-2.3) | 1.4 (1.0-2.1) |
|  | Other | 1.4 (0.8-2.6) | 1.4 (0.6-2.9) | 1.5 (0.7-3.1) |
| Parity and previous caesarean | |  |  |  |
|  | Nulliparous | 1.8 (1.5-2.1) | 1.6 (1.3-2.0) | 1.5 (1.1-1.9) |
|  | Parous without previous caesarean | Reference | Reference | Reference |
|  | Parous with previous caesarean | 1.3 (1.0-1.8) | 1.3 (0.9-1.8) | 1.3 (0.9-1.8) |
| Preexisting hypertension | | 0.9 (0.4-2.1) | 0.8 (0.4-1.9) | 0.6 (0.3-1.5) |
| Preexisting insulin-treated diabetes | | 1.8 (0.9-3.5) | 2.3 (1.2-4.6) | 1.7 (0.9-3.3) |
| Other preexisting chronic condition | | 1.1 (0.8-1.5) | 1.0 (0.8-1.4) | 1.1 (0.8-1.4) |
| Mode of conception | |  |  |  |
|  | Spontaneous | Reference | Reference | Reference |
|  | Ovulation-inducting drugs alone | 1.2 (0.9-1.6) | 1.1 (0.8-1.4) | 1.1 (0.8-1.4) |
|  | In vitro fertilization with autologous oocytes | 1.5 (1.2-1.8) | 1.3 (1.1-1.6) | 1.3 (1.0-1.6) |
|  | Oocyte donation | 2.6 (1.9-3.5) | 1.9 (1.4-2.7) | 1.6 (1.1-2.3) |
| Chorionicity | |  |  |  |
|  | Dichorionic | Reference | Reference | Reference |
|  | Monochorionic | 0.8 (0. 7-1.0) | 0.9 (0.7-1.2) | 0.9 (0.7-1.2) |
| Insulin-treated gestational diabetes | | 1.1 (0.8-1.7) | - | 1.0 (0.7-1.6) |
| Gestational hypertension | | 1.9 (1.4-2.5) | - | 1.2 (0.9-1.6) |
| Non-severe preeclampsia | | 2.6 (2.2-3.2) | - | 2.3 (1.8-2.9) |
| Placenta praevia | | 3.8 (2.5-5.9) | - | 3.5 (2.3-5.3) |
| Twin-to-twin transfusion syndrome | | 0.7 (0.4-1.3) | - | 0.9 (0.5-1.7) |
| Premature rupture of membranes | | 0.7 (0.5-1.0) | - | 0.7 (0.5-1.0) |
| Macrosomia | | 1.5 (1.1-2.0) | - | 2.0 (1.4-2.6) |
| Maternity hospital : | |  |  |  |
| Annual volume of twin deliveries | | |  |  |
|  | <50 | 0.8 (0.6-0.9) | - | 1.1 (0.7-1.6) |
|  | [50-99] | 0.8 (0.7-1.0) | - | 1.0 (0.8-1.2) |
|  | ≥100 | Reference | - | Reference |
| Level of care | |  |  |  |
|  | I | 0.3 (0.1-0.9) | - | 0.4 (0.1-1.4) |
|  | II | 0.8 (0.6-0.9) | - | 0.8 (0.5-1.1) |
|  | III | Reference | - | Reference |

RR, relative risk; CI, confidence interval

*Each relative risk is adjusted for all other variables in the table, multilevel multivariate Poisson regression model, with imputed data
