# Supplementary material for: Risk factors and high-risk subgroups of severe acute maternal morbidity in twin pregnancy: A population-based study
Source: PLoS One. 2020 Feb 28;15(2):e0229612. doi: 10.1371/journal.pone.0229612 (PMC7048407; doi:10.1371/journal.pone.0229612)
Supplement: S4 Table — RR, relative risk; CI, confidence interval *Each relative risk is adjusted for all other variables in the table, multilevel multivariate Poisson regression model. (DOC) [file pone.0229612.s004.doc]

**S4 Table. Risk factors for intrapartum and postpartum severe acute maternal morbidity in twin pregnancies, sensitivity analysis including delivery-related characteristics, JUMODA cohort (n=8**789 women)

| **Potential risk factors** | | **Crude RR** | **Adjusted RR*** |
| --- | --- | --- | --- |
|  |  | **(95% CI)** | **(95% CI)** |
| Maternal age (years) | |  |  |
|  | <30 | Reference | Reference |
|  | [30-35[ | 1.1 (0.9-1.3) | 1.1 (0.9-1.3) |
|  | [35-40[ | 0.8 (0.6-1.1) | 0.7 (0.6-1.0) |
|  | ≥40 | 1.6 (1.2-2.2) | 1.0 (0.7-1.5) |
| Body mass index before pregnancy (Kg.m-2) | | |  |
|  | <18.5 | 1.1 (0.8-1.5) | 1.2 (0.8-1.6) |
|  | [18.5-24.9] | Reference | Reference |
|  | [25-29.9] | 0.9 (0.7-1.1) | 0.8 (0.7-1.1) |
|  | [30-34.9] | 0.8 (0.6-1.1) | 0.7 (0.5-1.0) |
|  | ≥35 | 0.8 (0.5-1.2) | 0.8 (0.4-1.3) |
| Country of birth | |  |  |
|  | Europe | Reference | Reference |
|  | North Africa | 1.2 (0.9-1.5) | 1.3 (0.9-1.7) |
|  | Sub-Saharan Africa | 1.3 (0.9-1.9) | 1.5 (1.0-2.1) |
|  | Others | 1.5 (0.8-2.7) | 1.5 (0.7-3.0) |
| Parity and previous caesarean | |  |  |
|  | Nulliparous | 1.9 (1.5-2.3) | 1.5 (1.1-1.9) |
|  | Multiparous without previous caesarean | Reference | Reference |
|  | Multiparous with previous caesarean | 1.4 (1.0-1.9) | 1.2 (0.9-1.8) |
| Preexisting hypertension | | 1.3 (0.6-2.6) | 0.9 (0.4-1.8) |
| Preexisting insulin-treated diabetes | | 2.3 (1.2-4.6) | 1.7 (0.9-3.3) |
| Other preexisting chronic condition | | 1.1 (0.8-1.5) | 1.1 (0.8-1.5) |
| Mode of conception | |  |  |
|  | Spontaneous | Reference | Reference |
|  | Ovulation-inducting drugs alone | 1.2 (0.9-1.6) | 1.1 (0.8-1.5) |
|  | In vitro fertilization with autologous oocytes | 1.5 (1.2-1.8) | 1.3 (1.0-1.6) |
|  | Oocyte donation | 2.7 (2.0-3.7) | 1.7 (1.2-2.5) |
| Chorionicity | |  |  |
|  | Dichorionic | Reference | Reference |
|  | Monochorionic | 0.8 (0.7-1.0) | 1.0 (0.8-1.3) |
| Insulin-treated gestational diabetes | | 1.2 (0.8-1.8) | 1.1 (0.0-1.6) |
| Gestational hypertension | | 2.1 (1.7-2.8) | 1.2 (0.9-1.7) |
| Non-severe preeclampsia | | 2.9 (2.4-3.6) | 2.5 (1.9-3.2) |
| Placenta praevia | | 3.3 (2.0-5.5) | 3.1 (2.1-4.8) |
| Twin-to-twin transfusion syndrome | | 0.5 (0.2-1.0) | 0.7 (0.3-1.4) |
| Premature rupture of membranes | | 0.7 (0.5-0.9) | 0.8 (0.6-1.2) |
| Gestational age at delivery (weeks days) | | |  |
|  | <37 0/7 | 0.8 (0.7-1.0) | 0.8 (0.7-0.9) |
|  | ≥ 37 0/7 | Reference | Reference |
| Mode of delivery | |  |  |
|  | Vaginal for both twins | Reference | Reference |
|  | Caesarean before labor | 1.5 (1.3-1.9) | 1.3 (1.0-1.6) |
|  | Caesarean during labor | 2.1 (1.7-2.7) | 1.6 (1.2-2.1) |
| Macrosomia | | 1.4 (1.1-1.7) | 1.5 (1.2-1.9) |
| Maternity hospital: | |  |  |
| Annual volume of twin deliveries | | |  |
|  | <50 | 0.8 (0.6-0.9) | 1.1 (0.7-1.6) |
|  | [50-99] | 0.8 (0.7-1.0) | 1.0 (0.8-1.3) |
|  | ≥100 | Reference | Reference |
| Level of care | |  |  |
|  | I | 0.4 (0.2-1.1) | 0.5 (0.1-1.5) |
|  | II | 0.8 (0.6-0.9) | 0.7 (0.5-1.1) |
|  | III | Reference | Reference |

RR, relative risk; CI, confidence interval

*Each relative risk is adjusted for all other variables in the table, multilevel multivariate Poisson regression model
