# Supplementary material for: Risk factors and high-risk subgroups of severe acute maternal morbidity in twin pregnancy: A population-based study
Source: PLoS One. 2020 Feb 28;15(2):e0229612. doi: 10.1371/journal.pone.0229612 (PMC7048407; doi:10.1371/journal.pone.0229612)
Supplement: S1 Fig — (PPT) [file pone.0229612.s005.ppt]

## Slide 1
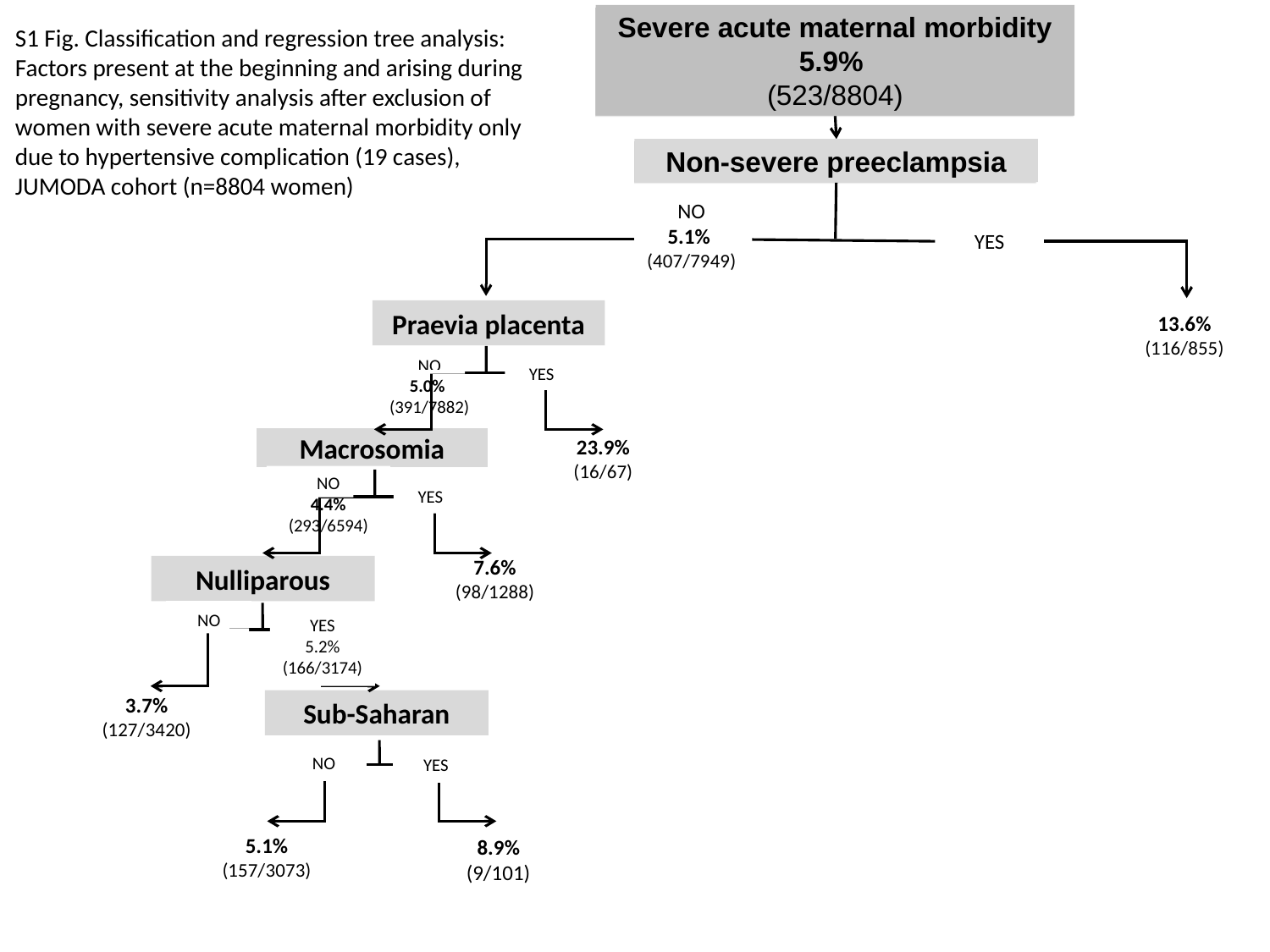

Severe acute maternal morbidity
5.9%
(523/8804)
Non-severe preeclampsia
YES
Praevia placenta
NO
5.0%
(391/7882)
Macrosomia
23.9%
(16/67)
NO
4.4%
(293/6594)
Nulliparous
NO
3.7%
(127/3420)
13.6%
(116/855)
S1 Fig. Classification and regression tree analysis: Factors present at the beginning and arising during pregnancy, sensitivity analysis after exclusion of women with severe acute maternal morbidity only due to hypertensive complication (19 cases), JUMODA cohort (n=8804 women)
NO
5.1%
(407/7949)
YES
YES
7.6%
(98/1288)
YES
5.2%
(166/3174)
Sub-Saharan
NO
YES
5.1%
(157/3073)
8.9%
(9/101)
