# Supplementary material for: Risk factors and high-risk subgroups of severe acute maternal morbidity in twin pregnancy: A population-based study
Source: PLoS One. 2020 Feb 28;15(2):e0229612. doi: 10.1371/journal.pone.0229612 (PMC7048407; doi:10.1371/journal.pone.0229612)
Supplement: Attachment — Submitted filename: response to Reviewers.docx [file pone.0229612.s006.docx]

Dr Diane Korb

INSERM U1153, Obstetric, Perinatal, and Pediatric Epidemiology Research Team

53 avenue de l’Observatoire, 75014 Paris, France

Tel: +33(0)1 42 34 55 80 Fax: +33 (0)1 43 26 89 79

Email: diane.korb@inserm.fr

Paris, December 20^th^ 2019

Dear Editor,

Thank you for your response on December 3^th^ 2019, concerning our manuscript PONE-D-19-27799 entitled “Risk factors and high-risk subgroups of severe acute maternal morbidity in twin pregnancy: a population-based study” informing us you would be willing to give further consideration to a revised version.

The authors are very grateful to the Reviewers and Editors for their constructive help. We think the paper has been much improved. Our revised version has taken into account all the following points raised by the Reviewers and Editors.

All the authors have read and approved the revised version of the paper.

We hope our manuscript now meets the standards of *Plos One*.

Yours sincerely,

Diane Korb

All line numbers refer to the revised version of the manuscript.

**Response to the Editor**

**In addition to the few issues raised by the reviewers, please use SI unit (convention units may be use in parenthesis). Also use English phrase for conditions such as “Placenta previa”.**

As requested, we replaced American by English language.

**1. Thank you for stating the following in the Acknowledgments Section of your manuscript:**

**Supported by a grant from the French Ministry of Health (Programme Hospitalier de Recherche Clinique, AOM2012)**

**We note that you have provided funding information that is not currently declared in your Funding Statement. However, funding information should not appear in the Acknowledgments section or other areas of your manuscript. We will only publish funding information present in the Funding Statement section of the online submission form.**

**Please remove any funding-related text from the manuscript and let us know how you would like to update your Funding Statement. Currently, your Funding Statement reads as follows:**

**The authors received no specific funding for this work**.

We modified the online submission form accordingly.

**2. Please provide additional details regarding participant consent. In the ethics statement in the Methods and online submission information, please ensure that you have specified (1) whether consent was informed and (2) what type you obtained (for instance, written or verbal). If your study included minors, state whether you obtained consent from parents or guardians. If the need for consent was waived by the ethics committee, please include this information.**

We precised in line 161: “They approved that this observational study waived the need to obtain written informed consent according to the French law.”

**3. PLOS requires an ORCID iD for the corresponding author in Editorial Manager on papers submitted after December 6th, 2016. Please ensure that you have an ORCID iD and that it is validated in Editorial Manager. To do this, go to ‘Update my Information’ (in the upper left-hand corner of the main menu), and click on the Fetch/Validate link next to the ORCID field. This will take you to the ORCID site and allow you to create a new iD or authenticate a pre-existing iD in Editorial Manager. Please see the following video for instructions on linking an ORCID iD to your Editorial Manager account:**

I added my ORCID iD in the online submission form (https://orcid.org/0000-0002-3074-8269).

**4. In your Data Availability statement, you have not specified where the minimal data set underlying the results described in your manuscript can be found. PLOS defines a study's minimal data set as the underlying data used to reach the conclusions drawn in the manuscript and any additional data required to replicate the reported study findings in their entirety. All PLOS journals require that the minimal data set be made fully available. For more information about our data policy, please see https://clicktime.symantec.com/3YZSF9bEtVTXuArrvN4RTeq6H2?u=http%3A%2F%2Fjournals.plos.org%2Fplosone%2Fs%2Fdata-availability.**

**Upon re-submitting your revised manuscript, please upload your study’s minimal underlying data set as either Supporting Information files or to a stable, public repository and include the relevant URLs, DOIs, or accession numbers within your revised cover letter. For a list of acceptable repositories, please see https://clicktime.symantec.com/34uYZEGrViwwJiXZ15MZ2ch6H2?u=http%3A%2F%2Fjournals.plos.org%2Fplosone%2Fs%2Fdata-availability%23loc-recommended-repositories. Any potentially identifying patient information must be fully anonymized.**

**Important: If there are ethical or legal restrictions to sharing your data publicly, please explain these restrictions in detail. Please see our guidelines for more information on what we consider unacceptable restrictions to publicly sharing data: https://clicktime.symantec.com/3VUWv2qA9KnXzUZXsdCzx4V6H2?u=http%3A%2F%2Fjournals.plos.org%2Fplosone%2Fs%2Fdata-availability%23loc-unacceptable-data-access-restrictions. Note that it is not acceptable for the authors to be the sole named individuals responsible for ensuring data access.**

**We will update your Data Availability statement to reflect the information you provide in your cover letter.**

The data underlying the findings cannot be made freely available because of ethical and legal restrictions. This is because the present study includes an important number of variables that, together, could be used to re-identify the participants based on a few key characteristics and then be used to have access to other personal data. Therefore, the French National Data Safety Authority (CNIL) strictly forbids making such data freely available. However, they can be obtained upon request from the JUMODA steering committee. Readers may contact: diane.korb@inserm.fr to request the data.

**5. One of the noted authors is a group or consortium: JUmeaux MODe d’Accouchement (JUMODA) study group and the Groupe de Recherche en Obstétrique et Gynécologie (GROG)**

**In addition to naming the author group, please list the individual authors and affiliations within this group in the acknowledgments section of your manuscript. Please also indicate clearly a lead author for this group along with a contact email address.**

Individual collaborators of JUMODA group and GROG are not co-authors of this manuscript.

We have added in the acknowledgments section the individual collaborators of JUMODA group and GROG and their affiliations. In supplementary file, we added the lead author for these groups along with contact email address.

**Response to the Reviewers**

**Reviewer #1: This manuscript uses a large population-based study of twin pregnancies "Jumonda" to identify risk factors for severe maternal morbidity and was planned prospectively. The primary outcome was a composite of including maternal death, severe hemorrhage (defined by need for second line therapy), transfusion >=units of pRBCs, uterine artery embolization, and several other severe adverse outcomes. They identified two subgroups of women with a >25% risk of developing severe acute maternal morbidity: those with both oocyte donation, non-severe preeclampsia, and nulliparas born in Sub-Saharan Africa with non-severe preeclampsia.**

We thank the Reviewer for his/her comment.

**Reviewer #2:**

**1. This is a potential wonderful paper. I am afraid I have major problems with the definition of severe morbidity.**

**Pre ecclampsia requiring delivery is certainly serious but NOT always a severe morbid event**.

We agree with the Reviewer that preeclampsia can reflect different steps within the morbidity continuum of hypertension-related complications.

The definition of severe preeclampsia in our study was the one retained in the EPIMOMS study. The EPIMOMS definition of severe maternal morbidity was obtained via an extensive national Delphi expert consensus process, with a panel of professionals including obstetricians, midwives, anaesthesiologists/intensive care specialists, and public health specialists. The process followed a Delphi-Rand design and consisted of two rounds followed by a final plenary session. The level of consensus for each round was fixed at 70%. Several published analyses were conducted with this definition confirming the recognition of its validity. (1-5)

In this multicriteria definition of severe *maternal* morbidity, severe preeclampsia can be defined through various criteria that indicate maternal : either a preeclampsia which indicated an induction of a preterm delivery before 32 gestational weeks *for a maternal indication;* or a preeclampsia associated with eclampsia, HELLP syndrome, or placental abruption; or responsible for a severe organ dysfunction (as per Epimoms specific definitions for each dysfunction): respiratory dysfunction, renal dysfunction, hepatic dysfunction.

To define severe (for the mother) preeclampsia apart from the aforementioned preeclampsia complications, criterion retained was the gestational age at induced delivery and not proteinuria or blood pressure cut-offs, because thresholds are not consensual. Severe preeclampsia remote from term is challenging and requires assessment of the benefit-risk balance for the fetus and for the mother between an expectant management and an induced preterm delivery. Before 32 gestational weeks if an induced preterm delivery was decided for a main maternal indication, this can only be for a severe event compromising maternal health allowing to consent for neonatal morbidity induced by prematurity.

Furthermore, we performed a sensitivity analysis after exclusion of severe acute maternal morbidity cases only due to hypertensive complications (n= 19 women) and our results were unchanged (S4A Table, and in below), as mentioned in the methods section line 137 to 142 and in the results section line 247 to 252.

Therefore, we believe that SAMM events due to severe preeclampsia were always severe morbid events and do not compromise the validity of our results.

Table S4A: Risk factors for severe acute maternal morbidity in twin pregnancies, sensitivity analysis after exclusion of women with severe acute maternal morbidity only due to hypertensive complication (19 cases), JUMODA cohort (n=8804 women)

|  | |  | **At the beginning of pregnancy** | **At the end of pregnancy** |
| --- | --- | --- | --- | --- |
| **Potential risk factors** | | **Crude RR** | **Adjusted RR** | **Adjusted RR*** |
|  |  | **(95% CI)** | **(95% CI)** | **(95% CI)** |
| Maternal age (years) | |  |  |  |
|  | <30 | Reference | Reference | Reference |
|  | [30-35[ | 1.0 (0.9-1.3) | 1.0 (0.9-1.3) | 1.0 (0.9-1.2) |
|  | [35-40[ | 0.8 (0.6-1.0) | 0.8 (0.6-1.0) | 0.7 (0.6-1.0) |
|  | ≥40 | 1.7 (1.3-2.3) | 1.3 (0.9-1.9) | 1.2 (0.9-1.7) |
| Body mass index before pregnancy (Kg.m-2) | | |  |  |
|  | <18,5 | 1.0 (0.8-1.5) | 1.0 (0.8-1.5) | 1.1 (0.8-1.6) |
|  | [18.5-24.9] | Reference | Reference | Reference |
|  | [25-29.9] | 0.9 (0.7-1.1) | 0.9 (0.7-1.1) | 0.8 (0.7-1.1) |
|  | [30-34.9] | 0.8 (0.5-1.1) | 0.8 (0.5-1.1) | 0.7 (0.5-1.0) |
|  | ≥35 | 0.7 (0.4-1.2) | 0.8 (0.4-1.4) | 0.7 (0.4-1.2) |
| Country of birth | |  |  |  |
|  | Europe | Reference | Reference | Reference |
|  | North Africa | 1.2 (0.9-1.5) | 1.3 (1.0-1.7) | 1.3 (1.0-1.7) |
|  | Sub-Saharan Africa | 1.3 (0.9-1.8) | 1.6 (1.1-2.3) | 1.4 (1.0-2.1) |
|  | Other | 1.4 (0.8-2.6) | 1.4 (0.6-2.9) | 1.5 (0.7-3.1) |
| Parity and previous caesarean | |  |  |  |
|  | Nulliparous | 1.8 (1.5-2.1) | 1.6 (1.3-2.0) | 1.5 (1.1-1.9) |
|  | Parous without previous caesarean | Reference | Reference | Reference |
|  | Parous with previous caesarean | 1.3 (1.0-1.8) | 1.3 (0.9-1.8) | 1.3 (0.9-1.8) |
| Preexisting hypertension | | 0.9 (0.4-2.1) | 0.8 (0.4-1.9) | 0.6 (0.3-1.5) |
| Preexisting insulin-treated diabetes | | 1.8 (0.9-3.5) | 2.3 (1.2-4.6) | 1.7 (0.9-3.3) |
| Other preexisting chronic condition | | 1.1 (0.8-1.5) | 1.0 (0.8-1.4) | 1.1 (0.8-1.4) |
| Mode of conception | |  |  |  |
|  | Spontaneous | Reference | Reference | Reference |
|  | Ovulation-inducting drugs alone | 1.2 (0.9-1.6) | 1.1 (0.8-1.4) | 1.1 (0.8-1.4) |
|  | In vitro fertilization with autologous oocytes | 1.5 (1.2-1.8) | 1.3 (1.1-1.6) | 1.3 (1.0-1.6) |
|  | Oocyte donation | 2.6 (1.9-3.5) | 1.9 (1.4-2.7) | 1.6 (1.1-2.3) |
| Chorionicity | |  |  |  |
|  | Dichorionic | Reference | Reference | Reference |
|  | Monochorionic | 0.8 (0. 7-1.0) | 0.9 (0.7-1.2) | 0.9 (0.7-1.2) |
| Insulin-treated gestational diabetes | | 1.1 (0.8-1.7) | - | 1.0 (0.7-1.6) |
| Gestational hypertension | | 1.9 (1.4-2.5) | - | 1.2 (0.9-1.6) |
| Non-severe preeclampsia | | 2.6 (2.2-3.2) | - | 2.3 (1.8-2.9) |
| Placenta praevia | | 3.8 (2.5-5.9) | - | 3.5 (2.3-5.3) |
| Twin-to-twin transfusion syndrome | | 0.7 (0.4-1.3) | - | 0.9 (0.5-1.7) |
| Premature preterm rupture of membranes | | 0.7 (0.5-1.0) | - | 0.7 (0.5-1.0) |
| Macrosomia | | 1.5 (1.1-2.0) | - | 2.0 (1.4-2.6) |
| Maternity hospital : | |  |  |  |
| Annual volume of twin deliveries | | |  |  |
|  | <50 | 0.8 (0.6-0.9) | - | 1.1 (0.7-1.6) |
|  | [50-99] | 0.8 (0.7-1.0) | - | 1.0 (0.8-1.2) |
|  | ≥100 | Reference | - | Reference |
| Level of care | |  |  |  |
|  | I | 0.3 (0.1-0.9) | - | 0.4 (0.1-1.4) |
|  | II | 0.8 (0.6-0.9) | - | 0.8 (0.5-1.1) |
|  | III | Reference | - | Reference |

RR, relative risk; CI, confidence interval

*Each relative risk is adjusted for all other variables in the table, multilevel multivariate Poisson regression model, with imputed data

**2. In a large network Criteria to admit to an ICU will not all be the same and thus potential for bias**.

We agree with the Reviewer that intensive care units (ICU) admission can depend on several factors, including the organization of the health care system and the accessibility and availability of ICU. (6,7) However, maternal admission to ICU has been considered by many authors as a ‘‘proxy’’ for reasonably assessing SAMM. (8-16) In France, rates, causes and severity of maternal admissions to intensive care units were analyzed in a recent study and showed a decrease in the rate of maternal ICU admissions from 2010 to 2014, but that the admitted mothers presented more severe clinical conditions, requiring more often resuscitation procedures and techniques provided in ICU. (17) These results suggest that admission to ICU increasingly reflects severe maternal morbidity.

Moreover in our study, among the 121 women admitted to ICU, only 16 did not have any other criterion of the composite definition of SAMM, and indications of admission to ICU confirm the severity of this event (table below).

Therefore, we do not believe that inclusion of the ICU admission criterion could have resulted in a potential bias.

Table: Indications of admission to ICU in JUMODA study

| Indications of admission to ICU (not exclusive) | N (%)  N=121 |
| --- | --- |
| Severe postpartum hemorrhage | 75 (62.0) |
| Eclampsia | 5 (4.1) |
| HELLP syndrom | 24 (19.8) |
| Other hypertensive complications | 7 (5.8) |
| Respiratory dysfunction | 5 (4.1) |
| Severe infection | 5 (4.1) |
| Pulmonary embolism | 3 (2.5) |
| Hepatic dysfunction | 3 (2.5) |
| Others | 10 (8.3) |

**3. This has the potential for a significant impact please try to find another channel is Tiffany and why except it if I am open even though yours was conducted using a a delfi**

**Can you be more strict in your defn of SMM and redo ? It will be more meaningful for everyone**

For the reasons provided in response to previous comments, we believe that the Epimoms definition of SAMM obtained through a national consensus of experts is valid to define events of severe maternal morbidity and that it is not necessary to reanalyze the data with a more strict definition of SAMM.

Our results will be unchanged with a definition of SAMM excluding preeclampsia and admission to ICU.

**References :**

(1) Siddiqui A, Azria E, Howell EA, Deneux-Tharaux C. Associations between maternal obesity and severe maternal morbidity: Findings from the French EPIMOMS population-based study. Paediatr Perinat Epidemiol. 2019 Jan;33:7-16

(2) Korb D., Goffinet A., Seco A., Chevret S., Deneux-Tharaux C., pour le groupe EPIMOMS,Risk of severe maternal morbidity associated with cesarean delivery and the role of maternal age: a population-based propensity score analysis. CMAJ 2019 April 1;191:E352-60

(3) Madar H, Goffinet F, Seco A, Rozenberg P, Dupont C, Deneux-Tharaux C. Severe Acute Maternal Morbidity in Twin Compared With Singleton Pregnancies. Obstet Gynecol. 2019 Jun;133:1141–50.

(4) Korb D., Deneux-Tharaux C., Seco A., Goffinet F., Schmitz T., Risk of Severe Acute Maternal Morbidity According to Planned Mode of Delivery in Twin Pregnancies. Obstet Gynecol 2018, 132, 647-655

(5) Le Ray C, Pelage L, Seco A, Bouvier-Colle M-H, Chantry AA, Deneux-Tharaux C. Risk of severe maternal morbidity associated with in vitro fertilization: a population-based study. BJOG Int J Obstet Gynaecol. 2019 Jul;126:1033-1041

(6) Chantry AA, Deneux-Tharaux C, Bonnet MP, Bouvier-Colle MH. Pregnancy related

ICU admissions in France: trends in rate and severity, 2006-2009. Crit Care Med 2015;43:78–86.

(7) DREES. La Statistique annuelle des etablissements (SAE), http://drees.solidarites-

sante.gouv.fr/etudes-et-statistiques/open-data/etablissements-desante-

sociaux-et-medico-sociaux/article/la-statistique-annuelle-des-etablissements-

sae

(8) Baskett TF, O’Connell CM. Severe obstetric maternal morbidity: a 15-year population-based study. J Obstet 2005;25:7–9.

(9) Madan I, Puri I, Jain NJ, Grotegut C, Nelson D, Dandolu V. Characteristics of obstetric intensive care unit admissions in New Jersey. JMatern Fetal Neonatal Med 2009;22:785–90.

(10) Pollock W, Rose L, Dennis CL. Pregnant and postpartum admissions to the intensive care unit: a systematic review. Intensive CareMed 2010;36:1465–74.

(11) Reichenheim ME, Zylbersztajn F, Moraes CL, Lobato G. Severe acute obstetric morbidity (near-miss): a review of the relative use of its diagnostic indicators. Arch Gynecol Obstet 2009;280:337–43.

(12) Senanayake H, Dias T, Jayawardena A. Maternal mortality and morbidity: epidemiology of intensive care admissions in pregnancy. Best Pract Res Clin Obstet 2013;27:811–20.

(13) Tuncalp O, Hindin MJ, Souza JP, Chou D, Say L. The prevalence of maternal near miss: a systematic review. BJOG 2012;119:653–61.

(14) Wanderer JP, Leffert LR, Mhyre JM, Kuklina EV, Callaghan WM, Bateman BT. Epidemiology of obstetric-related ICU admissions in Maryland: 1999–2008*. Crit Care Med 2013;41:1844–52.

(15) Zeeman GG. Obstetric critical care: a blueprint for improved outcomes. Crit Care Med 2006;34:S208–14.

(16) Zwart JJ, Dupuis JR, Richters A, Ory F, van Roosmalen J. Obstetric intensive care unit admission: a 2-year nationwide population-based cohort study. Intens Care Med 2010;36:256–63.

(17) Barry Y, Deneux-Tharaux C, Saucedo M, Goulet V, Guseva-Canu I, Regnault N, et al. Maternal admissions to intensive care units in France: Trends in rates, causes and severity from 2010 to 2014. Anaesth Crit Care Pain Med. 2019 Aug;38:363–9.
